# Manual verses automatic inline ventricular function assessment using MRI

**DOI:** 10.1186/1532-429X-13-S1-P354

**Published:** 2011-02-02

**Authors:** Marie Wasielewski, Asad A Usman, Peter J Weale, James Carr

**Affiliations:** 1 Northwestern University, Chicago, IL, USA; 2 Siemens Healthcare, Chicago, IL, USA

## Objective

To determine the reliability of inline ventricular function (VF) tracking with cardiac MRI compared to a manual technique.

## Background

Computer-based inline VF software can aid in the quantitative analysis of left ventricular (LV) function. Inline VF automatically locates the LV base and apex, detects endo- and epicardial contours in each cardiac slice and phase, and performs a volumetric calculation to determine ejection fraction (EF), end-diastolic volume (EDV), end-systolic volume (ESV). The method presents an opportunity to reduce data processing time and hence reporting time. We compare three methods of post-processing: automatic, semi-automatic, and manual.

## Method

A stack of 9-12 SA steady state free precession (SSFP) slices from the base of the myocardium to the apex were obtained on a 1.5 Tesla scanner (MAGNETOM Aera Siemens, Germany). Scanning was achieved using developmental cardiac Day Optimizing Throughput (DOT) engine technology. DOT technology uses landmark based guided scan planning to define long and short axis views. The mitral valve annulus is detected on long-axis cine SSFP images which are subsequently used in the automated analysis.

SA SSFP cine images with inline contours were loaded in Argus (Siemens Workstation, Germany). In the semi-automatic group, Argus appointed contours were manually corrected when more than 50% of the LV was visualized and adjusted to exclude areas of the outflow tract. The source stack of SA cines were also manually segmented and contoured. Results of all three methods were compared for EF, EDV, ESV, and measuring time.

## Results

**Figure 1:**
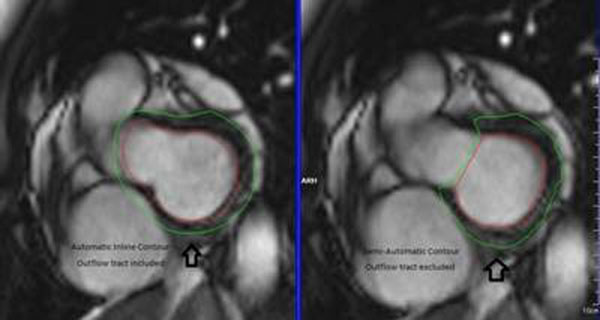


**Figure 2:**
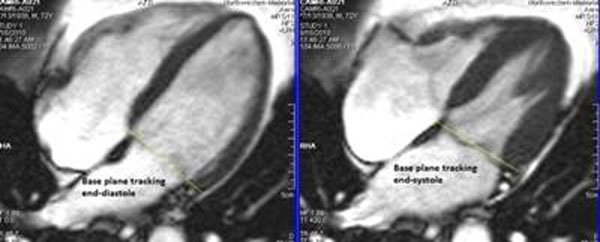


**Figure 3:**
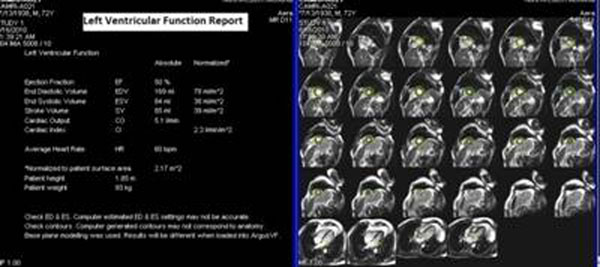


Complete results are presented in table 1. We scanned 20 healthy participants (41.8 ± 13 yrs). The mean automatic, semi-automatic, and manual EF was 53.8%, 66.1%, and 66.7%, respectively. The ESV across groups was 70.8, 49.7, and 48.6 ml, respectively. Both manual and semi-automatic techniques were statistically significantly different than the automatic technique for EF and ESV (p<0.05). However, the mean EDV across the groups was not statistically significantly different (p>0.05) 151.3, 144.8, and 143.7 ml, respectively. Figures 1, 2, 3

**Table 1:** 

Demographics			
**Mean Age (yrs)**	41.8 (± 13)		
**Gender**	8 Females & 12 Males		

**Results**			

**Technique**	***Automatic***	***Semi-Automatic***	***Manual***

**EF (%)**	53.8	66.1*	66.7*
**EDV (ml)**	151.3	144.8	143.7
**ESV (ml)**	70.8	49.7*	48.6*
**CO (l/min)**	4.9	5.78*	5.78*
**SV (ml)**	80.3	95.1*	95*
**Mean Measuring Time (min:sec)**	0	3:55*	5:55*

* ***Indicates p<0.05 statistical significance of group compared to automatic technique as a standard reference***

## Conclusion

We found that automated basal slice contours in end-systole can cause significant discrepancy in VF estimation. The decision to include the basal slice is the largest potential source of error in the accuracy of LV assessment. We suggest a semi-automated technique using a mix of automated and manual apical and basal correction of contours, to strike a balance between the precision of VF analysis and time when evaluating cardiac function.

